# *Cynara cardunculus* Crude Extract as a Powerful Natural Herbicide and Insight into the Mode of Action of Its Bioactive Molecules

**DOI:** 10.3390/biom10020209

**Published:** 2020-01-31

**Authors:** Sofiene Ben Kaab, Laurence Lins, Marwa Hanafi, Iness Bettaieb Rebey, Magali Deleu, Marie-Laure Fauconnier, Riadh Ksouri, M. Haissam Jijakli, Caroline De Clerck

**Affiliations:** 1 Integrated and Urban Plant Pathology Laboratory, Gembloux Agro-Bio Tech, University of Liège 2, Passage des Déportés, 2 5030 Gembloux, Belgium; marwa.hanafi@doct.uliege.be (M.H.); mh.jijakli@uliege.be (M.H.J.); 2 Laboratory of Aromatic and Medicinal Plants, Biotechnology Center at the Technopole of Borj-Cedria (CBBC), BP 901, 2050 Hammam-Lif, Tunisia; rosainess@yahoo.fr (I.B.R.); Ksouririadh@gmail.com (R.K.); 3 University of Tunis el Manar, Faculty of Mathematical, Physical and Natural Sciences of Tunis, 2092 Tunis, Tunisia; 4 Laboratory of Molecular Biophysics at Interfaces, Gembloux Agro-Bio Tech, University of Liège, Passage des Déportés, 2 5030 Gembloux, Belgium; L.lins@uliege.be (L.L.); magali.deleu@uliege.be (M.D.); 5 Laboratory of Chemistry of Natural Molecules, Gembloux Agro-Bio Tech, university of liege, Passage des Déportés, 2 5030 Gembloux, Belgium; marie-laure.fauconnier@ulg.ac.be

**Keywords:** bioactive compounds, oxidative stress, mode of action, weeds, herbicidal effect

## Abstract

The use of chemical herbicides could not only potentially induce negative impacts on the environment, animals, and human health, but also increase the weed resistance to herbicides. In this context, the use of plant extracts could be an interesting and natural alternative to chemical products. It is important to understand the mode of action of their bioactive compounds. This is why we have studied the herbicidal effect of *Cynara cardunculus* crude extract in terms of inhibition of weeds’ seedling growth and its impact on physiological parameters of treated plantlets, like conductivity, dry weight, and fluorescence, and biochemical parameters linked to oxidative stress. We have observed that *C. cardunculus* crude extract induces oxidative stress in the treated plants and consequently disturbs the physiological and biochemical functions of the plant cells. We have investigated the herbicidal activity of three bioactive compounds, naringenin, myricitrin, and quercetin, from the *C. cardunculus* crude extract. In both pre- and post-emergence trials, naringenin and myricitrin were significantly more phytotoxic than quercetin. We suggest that their differential initial interaction with the plant’s plasma membrane could be one of the main signals for electrolyte leakage and production of high levels of phenoxyl radicals.

## 1. Introduction

In the agricultural field, weeds are the first cause of yield reduction (about 32%), far ahead of pathogens (15%) [1]. They are constantly competing with crops for water, light, and nutrient resources, and therefore lead to huge economic losses [2]. Among the weed control methods, chemical herbicides are the most used ones. However, it is confirmed that their intensive use could induce negative impacts on the environment, animals, and human health, and increase the weed resistance to herbicides.

As an example, 210 species of weeds became resistant to herbicides in 2015 [3,4]. Moreover, it has been nearly 20 years that no herbicide with a new target site came into the market. It is, hence of primary importance to discover natural compounds with new herbicide target sites [5]. In this context, phenolic crude extracts containing several compounds could be a solution to combat weed resistances, as they usually have multisite action, which is not the case for synthetic herbicides [3].

Plant extracts, which were traditionally used for medical, nutritional, and even artisanal purposes [6] might be an alternative to develop natural herbicides for a sustainable agriculture [7]. They have been studied for many years for their fungicidal and bactericidal properties, but few studies have focused on their herbicidal properties [8].

*C. cardunculus* is known to be a good source of phenolic compounds and to have therapeutic potential as antidiabetic, antiproliferative, and antimicrobial agent, making it an interesting candidate for the development of future potential drugs [6,9]. Some genotypes of *C. cardunculus* are also very promising for biomass, grain, and oil production [10,11]. Moreover, a recent study showed that among 10 plant extracts, *C. cardunculus* crude methanolic extract had the best herbicidal activity in post-emergence [12].

The phytotoxic effect of this phenolic extract is probably due to the presence of an aromatic ring comprising several hydroxyl groups in its structure [10] but its mechanism of action is not clear yet [13]. One possibility is that it could disturb the plasma membrane and therefore cause the initial and basic effects related to oxidative stress. The latter could affect several cell functions, and finally leads to the destruction and death of cells [14,15,16,17,18]. The cuticle is considered as a first barrier for a molecule to penetrate into the plant, but little attention was given to the initial contact between these phenolic compounds and the plasma membrane. The latter is a complex dynamic entity.

The herbicidal activity of plant extracts has been already documented [19,20] but mostly consisted in determining the presence or absence of inhibitory effect and in characterizing the bioactive compounds. However, to our best knowledge, no study has focused on the effect of crude extracts and their individual bioactive molecules on the weeds. In this context, our study aims to characterize the physiological responses (such as conductivity, dry weight, fluorescence, and biochemical parameters linked to oxidative stress) of *Trifolium incarnatum* plants treated with *C. cardunculus* crude extract and their individual phenolic compounds compared to conventional herbicides in order to investigate their mode of action.

## 2. Material and Methods

### 2.1. Plant Material and Preparation of Formulated C. cardunculus Crude Extract

As described by [12], aerial parts of *C. cardunculus* were collected in its vegetative stage in March 2016 from Enfidha, located in the North of Tunisia. Phenolic extract of *C. cardunculus* was obtained by stirring 10 g of plant dry powder with 100 mL of methanol (Emplura EMD Millipore Corporation filiale de Merck KGaA) for 30 min. Methanol was then eliminated using a rotavapor in a vacuum at 45 °C, and residues were redissolved in a solution of tween 1%. Extract was kept for 24 h at 4 °C, filtered through a Whatman No 4 filter paper and stored at 4 °C. Afterwards, the extract was formulated following the method developed in [7] in order to ease the penetration of active molecules through epicuticular waxes.

### 2.2. Evaluation of the Herbicidal Effect of C. cardunculus Crude Extract and Its Bioactive Compounds

#### 2.2.1. Evaluation of Post-Emergence Activity of Whole Crude Extract under Greenhouse Conditions

Seeds of *T. incarnatum* were obtained from ECOSEM company in Belgium. The effect of formulated *C. cardunculus* extract was studied on 2–3-week-old plantlets grown under controlled conditions (natural photoperiod supplemented with artificial light if needed, temperature of 20 ± 3 °C according to the sunlight, and relative humidity of 60 ± 3%). The weeds’ seeds were sown in 11cm-diameter pots so that the whole surface of the ground was covered, and the plants were watered every day. Once the weeds reached the 2–3-leaf stage, several solutions were sprayed. They consisted of 10 mL of non-formulated *C. cardunculus* crude extract at 31 g L^−1^, formulated *C. cardunculus* crude extract at 31 g L^−1^, adjuvants used in the formulation alone (as negative control), and distilled water. In addition, commercial chemical and biological herbicides containing glyphosate at 7.5 g L^−1^ and pelargonic acid at 31 g L^−1^, respectively, were used as positive controls. Three replications were conducted for each treatment, in a completely randomized manner. The treated weed leaves were examined to assess several physiological parameters: dry weight, induced chlorophyll fluorescence, melondialdehyde (MDA), hydrogen peroxide production, and electrical conductivity.

##### Dry weight

The plants were collected 6 h or 3 days after the spraying, weighted and placed in an oven at 80 °C for 3 days, and then weighted again. The percentage of dry weight was calculated following Equation (1):

(1) $$\mathrm{Percentage}\;\mathrm{of}\;\mathrm{dry}\;\mathrm{weight}\;\left(\%\right)=\;\frac{\mathrm{weight}\;\mathrm{of}\;\mathrm{leaves}\;\mathrm{before}\;\mathrm{incubation}\;\left(6\;h\;\mathrm{or}\;3\;\mathrm{days}\right)}{\;\mathrm{weight}\;\mathrm{of}\;\mathrm{leaves}\;\mathrm{after}\;\mathrm{incubation}\;}\;$$

##### Induced Chlorophyll Fluorescence

Here again, treated leaves were collected 6 h or 3 days after spraying. The effect of the different herbicidal compounds on photosynthesis was tested using a HandyPEA fluorimeter (Hansatech Instruments, Pentney, Norfolk, UK). The instrument was set on Kinetic Mode and adjusted so that the initial Ft (instantaneous fluorescence signal) value in the control samples was approximately 210. The other parameters were optimized as described in [19].

##### Measurement of MDA

The melondialdehyde (MDA) is produced during lipid peroxidation and can be used as a marker of oxidative stress. Its concentration in the treated leaves was measured three days after the treatment using the protocol of [17] with some modifications. In brief, 100 mg of leaves treated with the herbicidal compounds were crushed in trichloroacetic acid (TCA) (10 mL, 0.1%, *w*/*v*) and centrifuged at 10 000× *g* for 10 min. One milliliter of the supernatant was added to 4 mL of thiobarbaturic acid (0.5%, *w*/*v*, in 20%, *w*/*v*, TCA). The mixture was heated at 95 °C for 30 min, cooled over ice, and then centrifuged at 10 000× *g* for 10 min. The absorbance of the supernatant was recorded at 532 nm and corrected for non-specific absorbance at 600 nm. The presence of MDA in the treated leaves is detected by a pink coloring. Its content was calculated using ε = 155 mM^−1^ cm^−1^ and expressed in nmol. g^−1^.

##### Measurement of electrical conductivity

Leaves of *T. incarnatum* collected for analysis three days after spraying were also examined for their electrical conductivity, as it is an indicator of cellular damage. The conductivity of each treated leaf was measured with a conductivity meter ( HACH HQ40d, Malines, Belgium).

##### Measurement of Hydrogen peroxide (H_2_O_2_)

Hydrogen peroxide (H_2_O_2_) is produced during oxidative stress. Its content in the treated leaves was determined using the protocol described in [18]. Treated leaves were collected after 3 days, extracted with trichloroacetic acid (TCA, 5 mL, 0.1%, *w*/*v*) in an ice bath, and the homogenate was centrifuged at 12,000× *g* for 15 min. In total, 0.5 mL of supernatant was then mixed with 0.5 mL phosphate buffer (pH 7) and 1 mL of potassium iodide (1 M). The absorbance of this reaction mixture was measured at 390 nm. Hydrogen peroxide content was determined using ε = 0.28 µM^−1^ cm^−1^, and expressed in nmol g^−1^.

#### 2.2.2. Evaluation of Post-Emergence Activity of Crude Extract’s Major Compounds under Greenhouse Conditions

Another experiment was performed to study the post-emergence activity of the major compounds contained in *C. cardunculus* crude extract: myricitin, quercetin, and naringenin [9]. These compounds were formulated in the same way as the whole crude extract, alone or in combination. The concentrations of the compounds were chosen to be in accordance with their concentration in the crude extract (myrcitrin at 60 µg mL^−1^, querecetin at 250 µg mL^−1^, and naringenin at 100 µg mL^−1^). Formulated myricitrin, quercetin, and naringenin alone and myrcitrin combination with querecetin at 250 µg mL^−1^ and naringenin at 100 µg mL^−1^ (Sigma, Brussels, Belgium) were sprayed on *T. incarnatum* plantlets at 2–3-leaf stage. The formulation compounds were also tested alone. Three repetitions were made in each case. Three days after spraying, the treated leaves of *T. incarnatum* were examined to assess wilting, necrosis, and chlorosis. The percentage of efficacy was calculated for each mix following Equation (2):

(2) Percentage of efficacy (%)= NT*100

where *N* refers to the number of necrotic or withered leaves, and *T* represents the total number of leaves.

#### 2.2.3. Evaluation of Pre-Emergence Activity under Laboratory Conditions

*T. incarnatum* seeds were sterilized using 0.5% sodium hypochlorite for 2 min. *C. cardunculus* crude extract was solubilized in tween 1% and then diluted with distilled water to the desired concentrations (0.75, 1.7, 3.4, 6.8, and 10 g L^−1^). Filter papers were placed in 11 cm diameter Petri dishes and moistened with 2 mL of Tween 1% solution only (which did not interfere with the different assays) for the negative control, and with *C. cardunculus* crude extract for the treated seedlings.

The major synthetic phenolic compounds of the crude extract (naringenin, myricitrin, and querecetin) (sigma, Brussels, Belgium) were tested individually under the same laboratory conditions in a separate experiment. They were solubilized in methanol 1% and then diluted with distilled water. The concentrations of these molecules were the same as in the post emergence experiment. In this case, filter paper was moistened with 2 mL of methanol 1% solution (which did not interfere with the experiment) as a negative control, and with the phenolic compound solutions for the treated seedlings.

For each treatment, ten seeds of *T. incarnatum* were placed in Petri dishes and three replicates (3 Petri dishes) were made for each solution. All Petri dishes for these experiments were randomly placed in a growth chamber at 23 ± 1 °C, in darkness. The hypocotyls and root lengths were measured after 7 days and the inhibition rate of the roots’ and hypocotyls’ length was calculated.

### 2.3. In Silico Interaction of Phenolic Compounds with Biomimetic Plant Plasma Membranes

**Figure 1:**
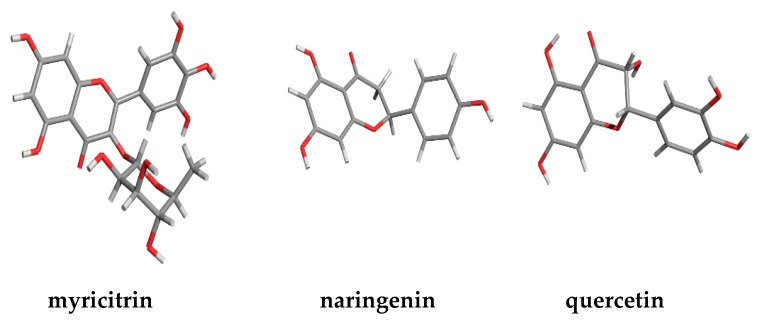
Chemical 3D structure of the three phenolic compounds studied.

The 3D structures of the phenolic compounds were constructed using HyperChem software (Hypercube, Inc., Gainesville, FL, USA) (Figure 1). The molecular geometry was optimized with the steepest-descent method using the MM+ force field, and a systematic analysis of the torsion angles using the structure tree method was performed as described previously in [21]. The most probable structure corresponding to the lowest conformational energy was used for further calculations.

The insertion of the molecule into an implicit bilayer was computed by the IMPALA procedure as described in [22]. Briefly, an implicit membrane is described as a continuous medium whose properties vary along the axis perpendicular to the bilayer plane (*z*-axis). The membrane properties are represented by energy restraints. The phenolic molecule is systematically moved along the *z*-axis by 1 Å steps, from one side of the membrane to the other and the restraints are calculated for each position.

### 2.4. Statistical Analysis

Statistical analysis was performed with Minitab^®^ Statistical Software (version 17, Minitab Inc., Paris, France). Results were examined statistically using one-way analysis of variance (ANOVA) followed by Tukey multiple range tests. The differences between individual means were considered significant if *p* < 0.05.

## 3. Results

### 3.1. Evaluation of Post-Emergence Activity under Greenhouse Conditions

**Figure 2:**
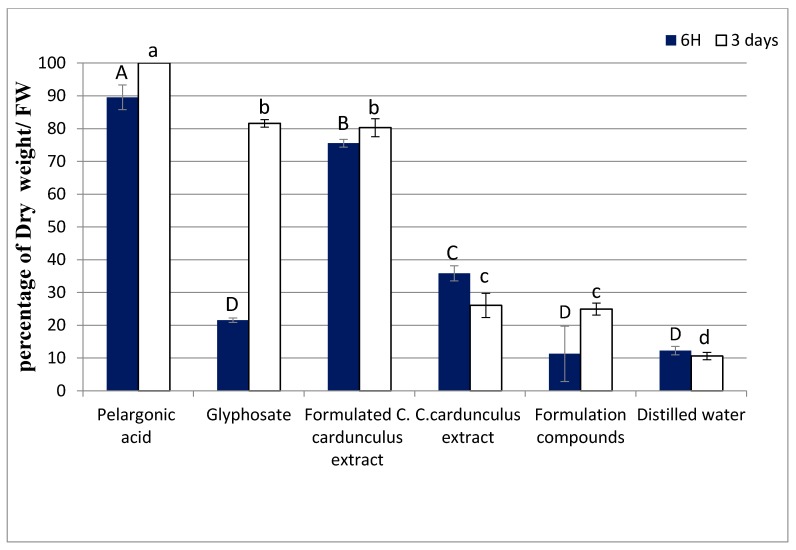
Percentage of dry weight of *Trifolium incarnatum* leaves 6 h and 3 days after the treatment. *C. cardunculus* crude methanolic extract with and without formulation at 31 g L^−1^, biological and chemical herbicide in the commercial concentration at the market (31 g L^−1^ and 7 g L^−1^, respectively), and herbicide based on pelargonic acid at 34 g L^−1^ (the same concentration as in the market), with formulation containing only vegetable oil and adjuvants. Values in a column followed by the same letter are not significantly different at *p* < 0.05, as established by Tukey’s test.

The post-emergence tests showed that the spraying of formulated *C. cardunculus* crude extract increased the leaves dry weight percentage of *T. incarnatum* compared with both negative control (formulation compounds and distilled water) and *C. cardunculus* without formulation (Figure 2).

**Figure 3:**
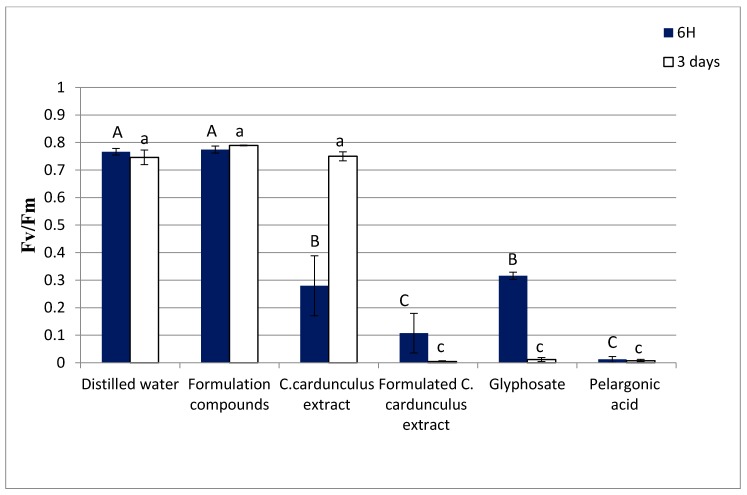
Fluorescence of leaves of *T. incarnatum* after 6 h and 3 days of treatment with *C. cardunculus* crude methanolic extract with and without formulation at 31 g L^−1^, fraction 2 with and without formulation at 20 g L^−1^ isolated from methanolic crude extract of *C. cardunculus* plant extract, biological and chemical herbicide in the commercial concentration at the market (31 g L^−1^ and 7.5 g L^−1^ respectively), and herbicide based on pelargonic acid at 34 g L^−1^ (the same concentration as in the market), with formulation containing only vegetable oil and adjuvant. Values in a column followed by the same letter are not significantly different at *p* < 0.05, as established by Tukey’s test.

The dry weight percentages of leaves treated by the formulated extract indeed reached 80%, while the dry weight percentages were 26% for the leaves treated with the non-formulated *C. cardunculus* extract and 24% for the formulation without extract. These results clearly show the positive effect of the formulation on the efficacy of the extract, enhancing its phytotoxic effects. On the other hand, we observed that pelargonic acid affected the dry weight more than formulated *C. cardunculus*. For glyphosate, the effect was significant after 3 days, but nothing can be detected within 6 h after spraying. The fluorescence of *T. incarnatum* leaves treated by formulated *C. cardunculus* crude extract was also measured (Figure 3).

Fluorescence analysis is often used in several studies to investigate the physiological aspects of photosynthesis and characterize plant photosynthetic performance [19].

The formulated crude extract of *C. cardunculus* decreased 90% of the leaves’ fluorescence even after 6 h in comparison with untreated leaves. On the other side, we observed that the extract without formulation does not affect the fluorescence after 3 days, showing once again that the formulation improved the efficacy of the extract. The statistical analysis showed that this effect is greater than that into the glyphosate treatment (diminution by 58% with regard to non-treated leaves).

In order to determine the oxidative response toward the herbicidal activity of formulated *C. cardunculus* extract, several parameters were assessed. MDA, conductivity, and H_2_O_2_ levels of *T. incarnatum* leaves sprayed with this extract were compared to the controls (Table 1). 

The results showed that formulated *C. cardunculus* extract significantly increases H_2_O_2_, conductivity levels, and MDA content in leaves compared with formulation without *C. cardunculus* extract, reaching 17.6 nmol g^−1^ MF^−1^, 961.5 µs cm^−1^, and 42.8 nmol g^−1^ MF^−1^, respectively. Statistical analysis indicated that H_2_O_2_ level in leaves treated with the formulated extract is higher than that in leaves treated with the commercial chemical herbicide. For conductivity and MDA, the effect was similar compared to the positive treatment. On the other hand, the co-formulants did not show any effect on this physiological parameter, assessing that the addition of these co-formulants does not affect the activity of *C. cardunculus* crude extract.

**Figure 4:**
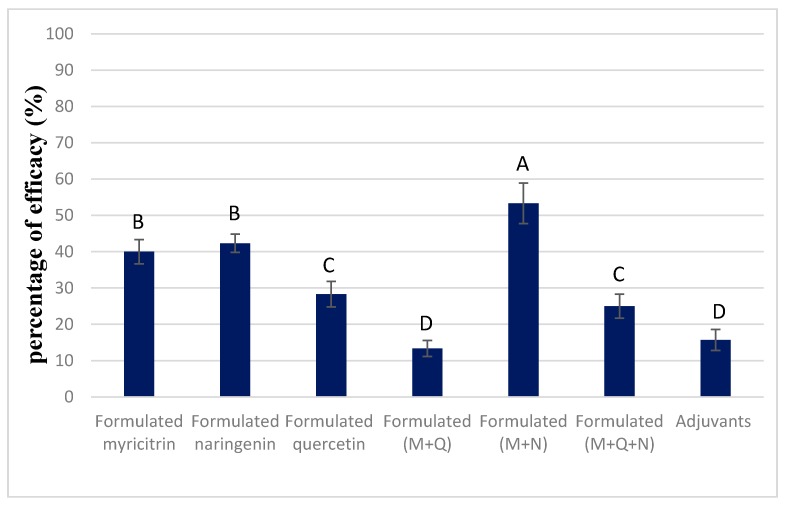
Post-emergence activity of formulated myricitrin at 60 µg mL^−1^ and its combination with quercetin at 250 µg mL^−1^ and naringenin at 100 µg mL^−1^ on *T. incarnatum* after 5 days. The formulation contained vegetable oil and adjuvants. Values in a column followed by the same letter are not significantly different at *p* < 0.05, as established by Tukey’s test.

The individual effects of the main compounds of our crude extract on *T. incarnatum* were also studied (Figure 4).

Myricitrin and naringenin showed phytotoxic effects on the leaves of *T. incarnatum*, causing a necrosis percentage of 40% and 42%, respectively, for treated leaves. Statistical analysis showed that these effects were significant compared with *T. incarnatum* treated by adjuvant only. In the case of quercetin, a lower herbicidal (28% of necrosis) was observed compared to the two other molecules. When combined, quercetin decreased the herbicidal effect of myricitrin by 13% while the combination with naringenin increased it by 11%.

### 3.2. Evaluation of Pre-Emergence Activity under Laboratory Conditions

**Figure 5:**
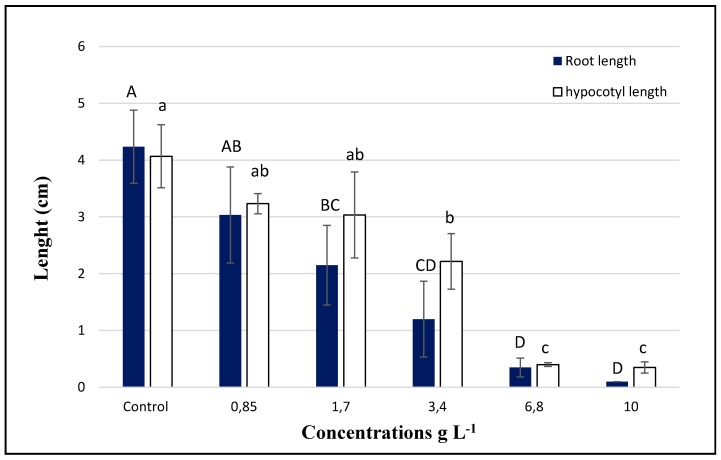
Phytotoxic effect of *C. cardunculus* crude extract in pre-emergence at different concentrations (0.85, 1.7, 3.4, 6.8, and 10 g L^−1^) on *T. incarnatum* seedling growth. Values in a column followed by the same letter are not significantly different at *p* < 0.05, as established by Tukey’s test.

**Figure 6:**
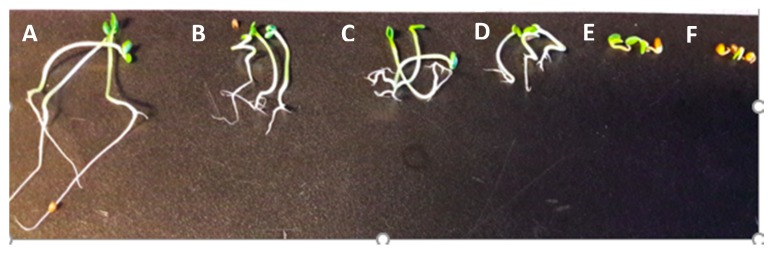
Inhibitory effects of *C. cardunculus* plant extract in pre emergence and on seedling growth of *T. incarnatum* at different concentrations (A, tween 1%; B, 0.85 g L^−1^; C, 1.7 g L^−1^; D, 3.4 g L^−1^; E, 6.8 g L^−1^; F, 10 g L^−1^).

The pre-emergence tests showed that the crude extract of *C. cardunculus* strongly inhibits the seedling growth of *T. incarnatum* (Figure 5 and Figure 6).

**Figure 7:**
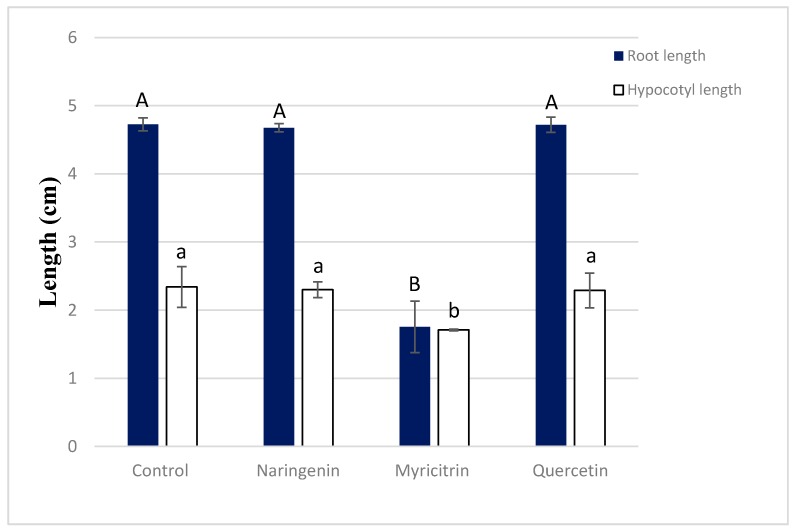
Pre-emergence activity of myricitrin at 60 μg mL^−1^, querecetin at 250 μg mL^−1^, and naringenin at 100 μg mL^−1^ on *T. incarnatum* after 5 days. Values in a column followed by the same letter are not significantly different at *p* < 0.05, as established by Tukey’s test.

The intensity of the effect varied with the concentrations applied. The inhibition reached up to 97% and 91%, respectively, for roots and hypocotyl growth after the application of *C. cardunculus* crude extract at 10 g L^−1^. In addition, the apparition of secondary roots was observed in the treated plants. Concerning the main compounds, only myricitrin decreased significantly the seedling growth of *T. incarnatum*, with a reduction of 62% for the roots and 26% for the hypocotyls (Figure 7). Statistical analysis showed that seeds treated by quercetin and naringenin have a similar development than the ones treated by distillated water.

## 4. Discussion

In our study, we have evaluated the herbicidal effect of *C. cardunculus* crude extract on *T. incarnatum* under laboratory conditions. It varied significantly according to the crude extract’s concentration, the maximal effect being obtained with the highest concentration, which is often the case in the literature [23,24]. The apparition of secondary roots for the treated seeds in pre-emergence tests could be caused by the interaction of the phenolic compounds from our extract with auxin transport [17], which affects the cell division and elongation processes during seed germination [25]. In addition, they could interact with enzymes involved in the mobilization of nutrients necessary for germination [26].

We have also studied the herbicidal effect of *C. cardunculus* crude extract in post-emergence, under greenhouse conditions by measuring physiological and biochemical parameters of treated *T. incarnatum*. The leaves showed signs of injury represented by several necrosis and chloroses, from 3 days after spraying on *T. incarnatum* leaves. These damages resulted in the increase of dry matter and of fluorescence observed in the leaves treated by the formulated extract. The fluorescence measurement has already been used in several studies to investigate the physiological aspects of photosynthesis and characterize plant’s photosynthetic performance [19]. For example, the study of [27] showed that *Eucalyptus globulus* aqueous extract affected the photochemical status of the treated plant. Indeed, the phenolic compounds in that extract (ferulic and p-hydroxybenzoic acids) decreased the chlorophyll fluorescence efficiency, represented by the ratio of variable to maximum fluorescence, Fv/Fm^−1^. This phenomenon was proven to be related to an inhibition of osmotically driven uptake of water under oxidative stress leading to a water content decrease and an increase in the percentage of the dry matter. In addition, we have confirmed that the formulation significantly improves the herbicidal effects of the extract. As described by [12], the formulation containing a vegetable oil and non-ionic surfactants increases their adsorption rate of active compounds, dissolves cuticular fatty acids, and therefore improves the penetration of the hydrophilic active substances [12,27,28].

The symptoms observed in our study could be linked to the formation of chlorophyllases (responsible of chlorophyll degradation and the change of thylakoid membrane structure) like it was observed by [29] studying the herbicidal effect of *Cupressus sempervirens*, *Juniperus phoenicea,* and *Tetraclinis articulata* extracts. Another possibility is that an oxidative stress caused an excess of energy, transferred to oxygen via chlorophyll and resulting in photo-oxidation damage. Excessive damage leads to membrane destruction and chlorophyll oxidation [30]. The measurement of the biochemical parameters (increase of MDA, H_2_O_2_ production, and lipid peroxidation levels) of treated *T. incarnatum* showed that our extract causes an oxidative stress that could initiate a sequence of reactions inducing damages in cellular organelles, ultimately leading to cell death [17]. For example, the H_2_O_2_ produced in mitochondria can react with the iron content to generate more reactive radicals like ^•^OH, damaging mitochondria and leading to reactive oxygen species (ROS) accumulation in the cells [20]. Lipid peroxidation originating from an oxidative degradation of the membrane lipids, produces MDA in various cell organelles and can therefore disrupt membrane integrity, induce oxidative phosphorylation, and inhibit the electron transfer chain [31]. Moreover, the high production of H_2_O_2_ could interfere with the activity of enzymes containing -SH groups, and therefore inhibit photosynthetic activity [32].

On the other hand, Sakihama et al., 2002 showed that they may induce the formation of ROS and consequently produce pro-oxidant compounds like phenoxy groups that could be detoxified by enzymatic and non-enzymatic reactions. However, under certain conditions (pH change, presence of ions such as Al, Zn, Mg, Cu, Fe, Cd, or Ca), these phenoxyl radicals can react with oxygen to generate H_2_O_2_, the hydroxyl radical (^•^OH), and a complex combination of semiquinones and quinones. The hydroxyl radical is known as the strongest oxidant and may cause enzyme inactivation, protein degradation, DNA damage, lipid peroxidation, and ultimately cell death [33,34].

On the other hand, the conductivity was increased in treated leaves of *T. incarnatum* in comparison to the control. It has been previously described by [35] that necrosis observed in treated leaves might be due to the leakage of ions and metabolites. This can be related to an alteration of the cell membrane and consequently cell death. On the other hand, flavonoids could inhibit electron transport in Photosystem II (PSII) and reduce the enzymatic activity of plastoquinone, which is an important coenzyme in the electron transfer chain during photosynthesis [36,37]. They strongly altered and degraded chloroplast ultrastructure of leaf parenchyma [38]. The high bioactivity of flavonoids can be linked to their ability to interact with membranes [39]. It has also been shown that naringenin can have a pro-oxidant effect on the lipids of membrane and could even cause cleavage of cell DNA via the production of phenoxyl radicals by forming complexes with transition metals [40]. They could hence damage cell membrane and alter metabolic functions [27]. The plasma membrane delimits the cell from its environment and has a fundamental function in perceiving the signals received from the outside to provide exchanges between the cytoplasm and the cellular environment [41,42]. As a consequence, any modification of the plant plasma membrane (PPM) structure by bioactive molecules will disturb its function and integrity, and hence affect the biochemical and physiological processes of the cell [43].

**Figure 8:**
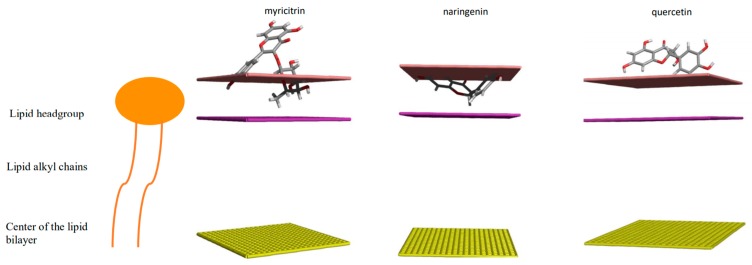
Three-dimensional representation of the optimal energy position for the interaction of flavonoids calculated by the IMPALA method. The pink line represents the outer medium/membrane interface, the mauve surface is the limit between the hydrophilic heads and the hydrophobic tails of the lipids, and the yellow surface represents the center of the membrane.

In that view, the phytotoxic effects that we observed could be linked to the fact that PPM is one of the targets of the individual compounds from the crude extract, due to their lipophilicity and their small size, as shown for some essential oil compounds [44]. To test this hypothesis, we used the in silico IMPALA method to predict the ability of molecules to penetrate into a model membrane (Figure 8). Quercetin does not penetrate into the membrane while naringenin and myricitrin could interact with the polar part of the membrane but seem to not be able to cross the apolar domain of the membrane. Since naringenin and myricitrin are more phytotoxic than quercetin, we can assume that the PPM could be involved in their biological activity, as shown for other natural compounds, such as gramin or hordenin [45].

In our study, we have also looked at the combined effects of the three major compounds of our crude extract. It was already shown that myricitrin, naringenin, and quercetin tested alone were phytotoxic compounds [9], but no study was presenting an herbicidal effect of mixed phenolic compounds to our best knowledge. It would, however, be really useful to have an herbicide combining several molecules with different mode of action, in order to avoid risks of resistance development.

We have hence observed that naringenin increases the herbicidal effect of myricitrin, making this combination a good candidate in an herbicide development. Similar studies about amine alkaloids [4] showed sarmentosine and sarmentine isolated from *Piper sarmentosum* have a high potential as a bioherbicide when combined together. In the same way, the mixtures of xanthoxyline and other natural compounds (alkene, phenolic aldehyde, and unsaturated fatty acid) in a Tween^®^ 80 solution have been proven to be able to significantly inhibit the germination and root growth of Chinese amaranth at lower concentrations [45]. All these studies confirmed that the synergistic effects of compounds could be applied for the development of potential herbicides with lower risk of resistance in weeds.

## 5. Conclusions

**Figure 9:**
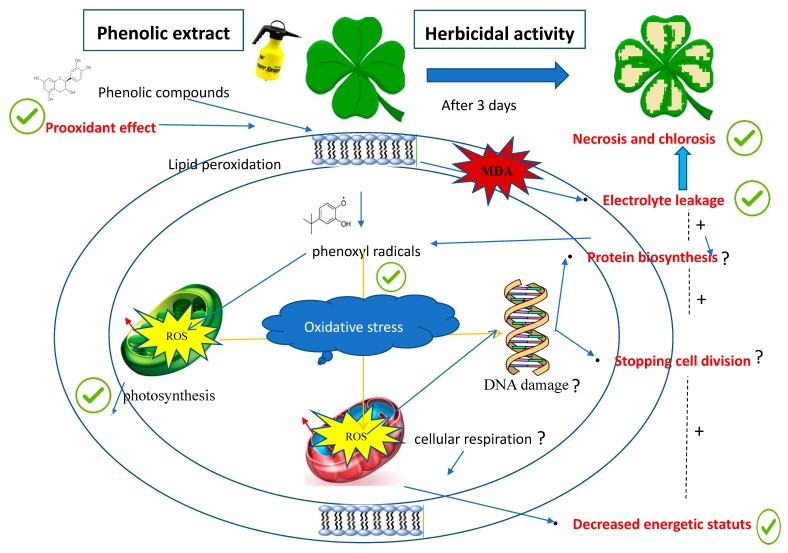
A hypothetical scheme for herbicidal effect mediated by phenolic compounds. Interaction of the phenolic compounds with the polar part of the membrane shown induces a prooxidant effect by forming phenoxyl radicals, which are very toxic for the cell. Phenoxyl radicals reduced photosynthetic activity by overproduction of the ROS.

Understanding the correlation between the mode of action of *C. cardunculus* crude extract and the oxidative stress seems to be one of the most challenging aspects of this study. The use of adjuvants could be a key role in the mode of action of our formulated extract. We can assume that the insertion of myricitrin and naringinin into the plasma membrane could be one of the main signals for electrolyte leakage and production of high levels of phenoxyl radicals. Moreover, the electrolyte leakage causes directly a disturbance in the electron transport chain in photosynthetic system, consequently increases ROS production, and decreases ATP levels (a source of energy in the cell). In this case, ROS can deactivate proteins, stop the cell division, and therefore cause cell death. All these modifications can cause many physiological processes that are observed in plants containing treated leaves. Among these modifications, we have noticed chloroses, necrosis, high percentage of dry weight, and the decrease of fluorescence. A hypothetical scheme for the mode of action for our phenolic compounds in plants is proposed in Figure 9.

On the other hand, our experiments shed some light on the effects of *C. cardunculus* individual bioactive components. An optimal formulation containing myricitrin and naringenin and having other modes of action than conventional herbicides, notably by potentially targeting the plasma membrane, could be highly useful to solve the herbicide resistance issue. Overall, *C. cardunculus* crude extract can be suggested as a potential eco-friendly herbicide and suitable source of natural herbicidal compounds.

## 6. Patents

Sofiene Ben kaab; Haissam Jijakli; Riadh Ksouri; Olivier Parisi; Simon Dal Mas. Herbicidal composition comprising at least one phenolic active compound. WO2019162388 (A1)—2019-08-29.

## Figures and Tables

**Table 1:** Physiological parameters of leaves treated with herbicidal compounds. The formulation contained vegetable oil and adjuvants. *Cynara cardunculus* extract was used at 31 g L^−1^ and glyphosate was used at its commercial concentration (7.5 g L^−1^).

Parameters	Distilled Water	Formulation Compounds	Formulated *C. cardunculus* Extract	Glyphosate
MDA (nmol g^−1^ FM^−1^)	4.95 ± 3.05 B	5.59 ± 5.21 B	42.80 ± 8.71 A	56.77 ±9.76 A
H2O2 (nmol g^−1^ FM^−1^)	1.18 ± 0.79 B	2.25 ±1.37 B	17.63 ± 2.13 A	4.91 ± 0.92 B
C (µs Cm^−1^)	188.20 ± 8.8 B	300.00 ± 13.33 B	961.50 ± 125.66 A	915.00 ± 33.33 A

Values in a column followed by the same letter are not significantly different at *p* < 0.05, as established by Tukey’s test.
